# Acute cerebrovascular events and inflammatory markers associated with COVID-19: An observational study

**DOI:** 10.25122/jml-2023-0283

**Published:** 2023-10

**Authors:** Merey Bakytzhanovna Jumagaliyeva, Dinmukhamed Nurniyazovich Ayaganov, Ibrahim Anwar Abdelazim, Samat Sagatovich Saparbayev, Nodira Miratalievna Tuychibaeva, Yergen Jumashevich Kurmambayev

**Affiliations:** 1 Department of Neurology, Psychiatry and Narcology, West Kazakhstan Marat Ospanov Medical University, Aktobe, Kazakhstan; 2 Department of Obstetrics and Gynecology, Faculty of Medicine Ain Shams University, Cairo, Egypt; 3 Department of Normal Physiology, West Kazakhstan Marat Ospanov Medical University, Aktobe, Kazakhstan; 4 Department of Neurology, Psychology and Psychotherapy, Tashkent Medical Academy, Tashkent, Uzbekistan

**Keywords:** cerebrovascular events, COVID-19, inflammatory markers, ACE2: Angiotensin-converting enzyme II, ASA: American Stroke Association, COPD: Chronic obstructive pulmonary disease, COVID-19: Coronavirus disease-19, CRP: C-reactive protein, CT: Computerized tomography, CVS: Cerebrovascular stroke, DM: Diabetes Mellitus, ESR: Erythrocyte sedimentation rate, GCS: Glasgow coma scale, ICH: Intracerebral hemorrhage, ICU: Intensive care unit, IL-6: Interleukin-6, IS: Ischemic stroke, LDH: Lactate dehydrogenase, MRC: Medical Research Council, MRI: Magnetic resonance imaging, NIHSS: National Institutes of Health Stroke Scale, PCR: Polymerase chain reaction, WKMU: West Kazakhstan Medical University

## Abstract

The novel Coronavirus disease (COVID-19) is associated with an increased risk of cerebrovascular events. About 1,228 cases of severe COVID-19 were hospitalized in the West Kazakhstan Medical University Hospital, in Aktobe, Kazakhstan, 1.22% (N=15) of whom were clinically diagnosed with acute cerebrovascular events and were included in the current study. COVID-19 was diagnosed using a nasopharyngeal polymerase chain reaction (PCR) test, blood count, inflammatory markers, and chest computerized tomography. The diagnosis of acute cerebrovascular events was based on the clinical manifestation. The participants’ data were reviewed to detect the prevalence of acute cerebrovascular events and the inflammatory markers associated with COVID-19 infection. The mean age of the participants was 66.9 years (±11.07), 53% (N=8) of them were male, while 47% (N=7) were female. Moreover, 13% (N=2) presented a history of cerebrovascular events, 87% (N=13) of the participants had hypertension, 47% (N=7) had coronary heart disease, 33% (N=5) had diabetes mellitus (DM), 13% (N=2) had cardiac arrhythmia, and 13% (N=2) had chronic obstructive pulmonary disease (COPD). The C-reactive protein was high in 100% (N=15) of participants, D-dimer in 87% (N=13) of them, and both the ferritin and interleukin-6 were high in 60% (N=9) of the participants. SARS-CoV-2 causes a systemic inflammatory response, and the presence of comorbidities increases the risk of acute cerebrovascular events in COVID-19-infected individuals. The elevated inflammatory markers in severely COVID-19-infected individuals support the inflammatory "cytokine storm" response theory.

## INTRODUCTION

In 2019, several cases of pneumonia were reported in Wuhan, China. The pneumonia was diagnosed later to be caused by a novel Coronavirus Disease (COVID-19) [1]. In February 2020, after the spread of the disease outside of China, the World Health Organisation (WHO) declared a COVID-19 outbreak in China [2]. COVID-19 caused thousands of deaths in each continent, and governments have actively adopted preventive measures to control its spread [3]. Respiratory symptoms are the main clinical manifestation of the infection, with interstitial pneumonia being showcased on chest X-ray or computerized tomography (CT) [4, 5]. Although the lungs are the main affected organ, SARS-CoV-2 is neurovirulent (a neuroinvasive virus), and about 36% of the infected individuals were hospitalized due to neurological manifestations [6]. Moreover, SARS-CoV-2 was associated with an increased risk of cerebrovascular events [7-9]. The American Stroke Association (ASA) recommends the screening of patients with acute stroke for COVID-19 infection [10-11]. Cerebrovascular stroke is the second cause of death worldwide with a mortality rate of about 5.5 million individuals per year [12]. Although ischemic stroke is the most common cerebrovascular complication of SARS-CoV-2, there were reported cases of hemorrhagic stroke and cerebral venous sinus thrombosis with SARS-CoV-2 [13]. The reported incidence of acute ischemic stroke with SARS-CoV-2 ranges from 2.5 to 5%, with most incidents being related to the occlusion of large vessels with embolic manifestation [13]. The neurological manifestation appears a few days after the onset of the symptoms or sometimes without COVID-19 symptoms (asymptomatic COVID-19) [13]. Consequently, the risk of cerebrovascular events in COVID-19-infected individuals is worth considering. This study aimed to detect the prevalence of acute cerebrovascular events and the inflammatory markers associated with COVID-19.

## MATERIAL AND METHODS

This observational cohort study was conducted on 1,228 cases of severe COVID-19 hospitalized patients at the West Kazakhstan Medical University Hospital in Aktobe, Kazakhstan. Out of all patients, 1.22% (N=15) were clinically diagnosed with acute cerebrovascular events and were included in the current study.

Adults (>18 years old) with confirmed COVID-19 infection who developed signs of acute cerebrovascular events (confirmed by two independent neurologists) with the onset of COVID-19 a few days after hospitalization were included. Adults with unconfirmed COVID-19 infection, who required brain magnetic resonance imaging (MRI) or computerized tomography (CT) to confirm the diagnosis of acute cerebrovascular events, or refused to participate were excluded from the sample.

The diagnosis of COVID-19 infection in the participants was based on the nasopharyngeal swab PCR test, lymphopenia in blood count, increased inflammatory markers [such as C-reactive proteins (CRP), D-Dimer, ferritin, and interleukin-6 (IL-6)], and chest CT.

Acute cerebrovascular events include ischemic stroke (IS), transient ischemic attack, intracerebral hemorrhage (ICH), subarachnoid hemorrhage, and cerebral venous sinus thrombosis [13].

Neuroimaging methods, including CT and MRI, are the gold standards for diagnosing acute cerebrovascular events, presenting>95% accuracy. Unfortunately, the participants were critically ill and no brain CT and/or MRI was used, and the diagnosis of acute cerebrovascular events was based on the clinical diagnosis by two independent neurologists.

The following participants' data were collected:

1. Age, gender, comorbidities, acute cerebrovascular events risk factors (i.e., hypertension, hypercholesterolemia, diabetes, heart diseases, contraceptive pills, and/or smoking habits), time from the onset of COVID-19 symptoms to the development of acute cerebrovascular events signs, days of stay in the intensive care unit (ICU), and mortality.
2. Laboratory results including the blood count and inflammatory markers [erythrocyte sedimentation rate (ESR), CRP, D-Dimer, ferritin, and IL-6].
3. Neurological assessment based on either the Glasgow Coma Scale (GCS), the National Institutes of Health Stroke Scale (NIHSS), or the Medical Research Council (MRC).

The participants’ data were reviewed to detect the prevalence of acute cerebrovascular events and the inflammatory markers associated with COVID-19.

## RESULTS

Out of the 1,228 cases of severe COVID-19 hospitalized, 1.22% (N=15) were clinically diagnosed with acute cerebrovascular events and were included in the current observational study.

### Participants’ characteristics and comorbidities

**Figure 1:**
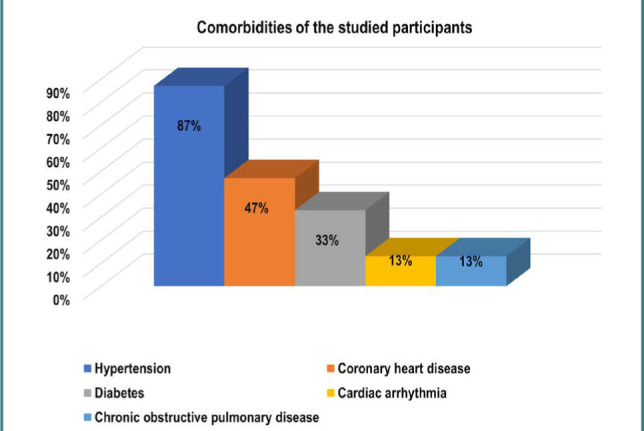
Prevalence of comorbidities in participants with acute cerebrovascular events

The mean age of the participants was 66.9 years (±11.07), 53% of them (N=8) were female, 47% (N=7) were male, 27% (N=4) were smokers, and 13% (N=2) had a previous history of cerebrovascular events. About 87% (N=13) of the participants had hypertension, 47% (N=7) had coronary heart disease, 33% (N=5) had DM, 13% (N=2) had cardiac arrhythmia, and 13% (N=2) had chronic obstructive pulmonary disease (COPD) (Table 1, Figure 1).

**Table 1:** Characteristics of the studied participants

No.	Gender	Age (Years)	Co-morbidities	Smoking	Time from the onset of Covid-19 symptoms to the development of ACA	Days of the stay in the ICU	Mortality
1	Male	71	Hypertension Atrial fibrillation COPD	-	1 day	14	-
2	Female	88	Hypertension	-	1 day	0	+
3	Female	67	DM and CHD	-	3 days	7	-
4	Female	66	Hypertension	-	6 days	3	+
5	Female	70	Hypertension and CHD	-	4 days	6	+
6	Male	54	DM - Hypertension Tuberculosis	Yes	10 days	14	+
7	Male	62	Hypertension	-	1 day	4	+
8	Male	71	Hypertension	Yes	1 day	5	-
9	Female	77	Hypertension and COPD	-	1 day	9	-
10	Female	66	Hypertension CHF and CHD	-	1 day	2	+
11	Female	66	DM - Hypertension CHD - Chronic renal disease	-	7 days	4	+
12	Male	50	Hypertension DM - CHD	Yes	7 days		
13	Male	65	Hypertension DM - CHD	-	5 days	2	+
14	Female	85	Hypertension Atrial fibrillation CHD	-	1 day	1	+
15	Male	46	-	Yes	1 day	11	-

CHD: Coronary heart disease; CHF: Chronic heart failure; COPD: Chronic obstructive pulmonary disease; DM: Diabetes mellitus; ICU: Intensive care unit

### Causes of the participants’ hospitalization

The signs of cerebrovascular events were the cause of hospitalization in 53% (N=8) of the participants, while 47% (N=7) were hospitalized because of severe COVID-19 clinical manifestations, and developed signs of acute cerebrovascular events a few days after hospitalization. The signs of acute cerebrovascular events developed within six days (range 3-10 days) after the severe COVID-19 symptoms in 47% (N=7) of the sample. Of all the participants with acute cerebrovascular events who were admitted to the ICU, 60% (N=9) deteriorated, presenting lower oxygen saturation, and eventually died despite extensive medical efforts and support within an average of 3.3 days after ICU admission (range 0-10 days) (Table 1).

### Participants’ laboratory results

**Figure 2:**
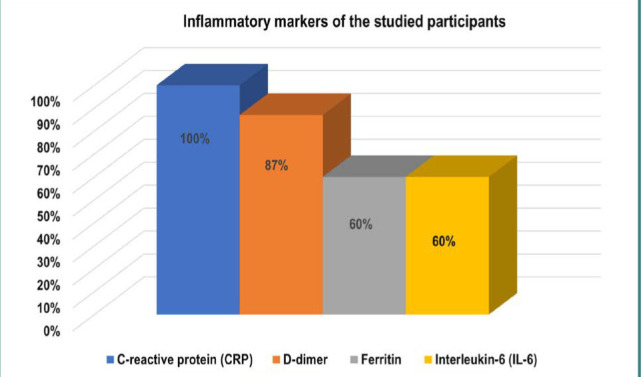
Prevalence of inflammatory markers in participants with severe COVID-19

The blood count showed leukocytosis, lymphopenia (characteristics of the COVID-19 infection), and increased inflammatory markers, namely CRP, D-dimer, ferritin, and IL-6. The CRP was high in 100% (N=15), D-dimer was high in 87% (N=13) of patients and both the ferritin and IL-6 were high in 60% (N=9) of the participants (Table 2 & Figure 2). The neurological assessment of the participants showed III-degree coma (GCS 5-6) in 20% (N=3) and sopor (GCS 9-10) in 27% (N=4). The mean (±SD) NIHSS score was 12.9 (±4.25). Poor functional status [3-6 on Medical Research Council (MRC)] was reported in 73% (N=11) of the sample (Table 3).

**Table 2:** Laboratory results of the participants, including the blood picture, inflammatory markers, and coagulation profile

	Blood picture				Inflammatory markers					Coagulation profile			
No.	Hb (gm%)	Lymphocytes (%)	Leukocytes X 10^9^/L	Platelets X 1000/L	ESR (mm/h)	CRP (mg/L)	D-dimer (ng/mL)	Ferritin (ug/L)	IL-6	APPT (Sec.)	INR	PTT (Sec.)	TT (Sec.)
1	157	38	9.2	332	6	10	0.34	40.4	18.4	31	1.25	88	16
2	135	6	9.8	124	26	127.9	3.05	350	27.5	27	1.06	97	18
3	108	12	6.3	229	53	61.9	1.1	72.2	16.9	3	1.18	93	16
4	142	5	27.6	119	8	45	0.88	350	28.9	41	1.28	85	22
5	144	11	5.7	145	31	39.9	0.85	206	25.8	31	1.25	94	18
6	163	16.5	4.4	136	10	11.4	0.2	14.8	16.7	27	1.1	99	22
7	153	23	11	563	15	112	9.25	45.9	38.9	32	1.32	83	12
8	134	16.7	15	170	13	39.2	2.5	1544	20.4	36	1.18	93	12
9	145	49.5	3.7	191	15	22.2	1.4	161	14.0	41	1.28	85	22
10	165	5	17.5	150	4	10	10	80	19.7	34	1.53	72	12
11	124	15	14	336	20	96	10	147.9	24.5	24	1.19	91	11
12	140	13.4	6.7	395	20	15.6	0.6	818.6	12.9	27	1.1	99	12
13	125	5	13.7	337	46	147.4	0.67	350	14.7	37	1.04	105	20
14	161	7	6	96.4	2	59	2.5	115.6	45.5	34	1.5	73	13
15	138	15.6	17	200	6	14.4	1514	350	20.6	32	1.31	76	19

APPT: Activated partial thromboplastin time; CRP: C-Reactive protein; ESR: Erythrocyte sedimentation rate; Hb: Hemoglobin; IL-6: Interlukein-6; INR: international normalized ratio; PTT: Partial thromboplastin time; TT: Thrombin time

**Table 3:** The studied participants neurological status

No.	Diagnosis	GCS	NIHSS	VAS	MRC	Modified Rankin Scale
1	Right middle cerebral artery-CVE (Dysphasia)	15	8	7	4/5 4/5	2
2	Right middle cerebral artery-CVE (Left-sided hemiparesis)	9	14	-	2/5 3/5	4
3	Left middle cerebral artery-CVE (Motor aphasia)	15	16	6	0/5 0/5	5
4	Right middle cerebral artery-CVE (Left-sided hemiplegia)	9	16	-	0/5 0/5	5
5	Left middle cerebral artery-CVE (Sensor and motor aphasia)	15	8	7	3/5 3/5	3
6	Right middle cerebral artery-CVE (Left-sided hemiplegia)	10	18	-	0/5 0/5	5
7	Left middle cerebral artery-CVE (Right-sided hemiplegia and motor aphasia)	9	18	-	0/5 0/5	5
8	Vertebrobasilar basin-CVE (Alternating Wallenberg syndrome)	15	4	8	5/5 5/5	1
9	Left middle cerebral artery-CVE (Right-sided mild hemiparesis)	15	10	4	4/5 4/5	2
10	Stem-CVE (Quadriplegia)	5	16	-	0/5 0/5	5
11	Vertebrobasilar basin-CVE (Brainstem syndrome)	5	16	-	0/5 0/5	5
12	Right middle cerebral artery-CVE (Right-sided hemiparesis and dysarthria)	15	10	8	4/5 3/5	2
13	Left middle cerebral artery-CVE (Left-sided hemiplegia)	15	10	5	0/5 2/5	5
14	Vertebrobasilar basin-CVE (Brainstem syndrome)	5	18	-	0/5 0/5	5
15	Left middle cerebral artery-CVE (Right-sided hemiparesis)	15	12	8	3/5 3/5	3

CVE: Cerebrovascular event; GCS: Glasgow Coma Scale; NIHSS: Stroke Scale of the National Institutes of Health; VAS: Visual Analog Scale; MRC: Medical Research Council

## DISCUSSION

The novel Coronavirus disease (COVID-19) was associated with an increased risk of cerebrovascular events. Consequently, this study aimed to detect the prevalence of acute cerebrovascular events and the inflammatory markers associated with COVID-19. The mean age of the studied participants was 66.9 years (±11.07), 53% of them were female, 47% were male and 13% had a previous history of cerebrovascular events. The risk of acute cerebrovascular events in COVID-19-infected individuals increased seven times after the age of 50 years with minimal or even without COVID-19 manifestations [14, 15]. The previous studies reported positive COVID-19 PCR in 18.4% of men compared to 13.3% of women and explained this finding by the presence of the male androgen hormone and/or the low level of COVID-19 antibodies in men [16-18]. About 87% of the participants had hypertension, 47% had coronary heart disease, 33% had DM, 13% had cardiac arrhythmia and 13% had COPD.

A meta-analysis of 46,248 COVID-19-infected participants showed that hypertension (17%), DM (8%), and cardiovascular diseases (5%) were the most common comorbidities or the most common risk factors for acute cerebrovascular events with COVID-19 [15]. SARS-CoV-2 plays a role in the development of diffuse vasculopathy or vasculitis following the endothelial cells and inflammatory cell infiltration with subsequent platelet aggregation and thrombosis [11]. Moreover, the COVID-19 infection is associated with hyperthermia and dehydration which predispose the body to endothelial damage, vasculopathy, and thrombosis [17, 18]. In this study, the clinical manifestation of acute cerebrovascular events developed within six days (range 3-10 days) after the severe COVID-19 symptoms in 47% of the participants. Acute cerebrovascular events are more common with severe COVID-19 infection and were reported in 5.7% of patients with severe COVID-19 infection and only in 0.8% of patients with mild COVID-19 [14, 19]. The blood panel of the participants showed leukocytosis, with lymphopenia (characteristics of the COVID-19 infection) and increased inflammatory markers CRP, D-dimer, ferritin, and IL-6. Increased D-dimer and CRP in severe COVID-19 and cerebrovascular events are constant findings with virus-associated microangiopathy [14, 19]. The SARS-CoV-2 virus induces a surge of inflammatory cytokines known as "cytokine storm" response [20]. The IL-6 is the primary "cytokine storm" component [21]. Zendelovska *et al*. [22], found the IL-6, interferon-γ, and endothelial growth factor (EGF) were significantly higher in COVID-19 infected compared to non-infected individuals. Additionally, the IL-1 (Anakinra) [23], and the IL-6 (Tocilizumab) [24] receptor blockers showed a significant improvement in COVID-19-infected individuals, which supports the COVID-19 "cytokine storm" response theory. The cerebrovascular events can be caused by the SARS-CoV-2 either directly due to neurovirulence (neuroinvasiveness) with a direct tropism to endothelial cells, or indirectly through the systemic inflammatory response "cytokine storm", endothelial damage, or vasculopathy, with subsequent microangiopathy and thrombosis. Shen *et al*. [19], found that COVID-19-associated ischemic stroke is usually preceded by COVID-19-related coagulopathy and thrombosis of the large vessels [19]. The renin-angiotensin system plays a critical role in the hypercoagulation status and thrombosis development. Moreover, the activation of the renin-angiotensin system is associated with increased angiotensin-II concentration with subsequent activation of the inflammatory cytokines "cytokine storm" in the vascular endothelium, vasculopathy, and thrombosis [25]. The elevated inflammatory markers CRP, IL-6, and D-dimer in most of the COVID-19-infected individuals support the inflammatory "cytokine storm" response theory and hypercoagulation status in severe COVID-19-infected individuals.

Moreover, the binding of the SARS-CoV-2 to the angiotensin-converting enzyme receptors causes a subsequent decrease in the angiotensin-converting enzyme-II (ACE2), abnormally high blood pressure, and increased risk of hemorrhagic stroke. Additionally, SARS-CoV-2 may trigger the cytokines cascade or "cytokine storm", which can worsen ischemic brain damage and increase the risk of cerebral hemorrhage [14, 26].

Moreover, pulmonary thrombosis and multiple organ failure were also reported with COVID-19 infection [15]. This observational cohort study was the first study conducted in the Republic of Kazakhstan to support the increased risk of acute cerebrovascular events in COVID-19-infected individuals and the COVID-19 inflammatory "cytokine storm" response theory.

The diagnosis of acute cerebrovascular events based on the clinical manifestations, and the absence of neuroimaging to confirm the diagnosis of acute cerebrovascular events was one of the limitations of this study (i.e., the studied participants were critically ill). The small sample size of participants admitted with severe COVID-19 infection was another limitation of this study.

## CONCLUSION

Certain comorbidities increase the risk of acute cerebrovascular events in COVID-19-infected individuals. The study revealed that 87% of the patients with acute cerebrovascular events had hypertension, 47% had coronary heart disease, 33% had DM, 13% had cardiac arrhythmia, and 13% had COPD. The elevated inflammatory markers including CRP, D-dimer, ferritin, and IL-6 in severe COVID-19 infected individuals support the inflammatory "cytokine storm" response theory.
